# Parental psychological control and cyberbullying among adolescents: the mediating roles of sleep quality and moral disengagement and the moderating role of grade

**DOI:** 10.3389/fpsyg.2025.1664970

**Published:** 2025-10-23

**Authors:** Jing Xu, Shufen Dai, Suo Jiang, Hui Xu

**Affiliations:** ^1^School of Mental Health, Wenzhou Medical University, Wenzhou, China; ^2^Chenzhou Vocational Technical College, Chenzhou, China; ^3^Institute of Medical Humanities, Wenzhou Medical University, Wenzhou, China; ^4^Wenzhou Key Laboratory of Basic and Translational Research for Mental Disorders, Wenzhou, Zhejiang, China

**Keywords:** parental psychological control, cyberbullying, sleep quality, moral disengagement, moderated chain mediation model

## Abstract

**Background:**

This study investigated how parental psychological control (PPC) influences adolescents’ cyberbullying behaviors through the mediating roles of sleep quality and moral disengagement, as well as examined the moderating effect of grade level.

**Methods:**

In this study, a total of 688 students from 23 schools in one province were enrolled, and they have completed several psychological assessments including PPC, sleep quality, moral disengagement, and cyberbullying. A moderated chain mediation model was conducted to examine their relationships.

**Results:**

The finding revealed that PPC significantly positive associated with adolescents’ engagement in cyberbullying. Mediation analyses demonstrated that both sleep quality and moral disengagement served as significant mediators in this relationship, and a sequential (chain) mediation effect was also observed. Furthermore, the effect of moral disengagement on cyberbullying was found to be moderated by grade level, and the relationship was stronger among highschoolers than pupils.

**Conclusion:**

These findings underscored the importance of parenting style, adolescent sleep health, and moral cognition in understanding and preventing cyberbullying, and suggested that effective intervention should address parental control strategies, promote healthy sleep habits, and enhance adolescents’ moral reasoning to reduce cyberbullying and support healthy adolescent development.

## 1 Introduction

Bullying behavior has become an increasingly severe social issue. Traditionally, bullying in the form of physical attacks, verbal insults or social exclusion has posed a threat to the mental health and social well-being of adolescents for a long time (Olweus, 2013; Smith, 2016). Unlike traditional bullying, cyberbullying is characterized by omnipresence, and it can reach victims whenever and wherever, which has a more profound impact due to anonymity and extensive dissemination capabilities (Smith and Slonje, 2009). Previous research has indicated that cyberbullying is closely associated with mental health issues such as depression and anxiety, and may lead to sleep disorders and a higher risk of suicide (Kowalski et al., 2014; Wang and Ngai, 2020). The incidence rate of cyberbullying has shown an upward trend globally, especially among children and adolescents (Falla et al., 2023; Yang et al., 2022). It is urgent to have the need for a better understanding of the mechanisms underlying cyberbullying.

The family environment is a crucial external factor affecting adolescent growth and may play a pivotal role in cyberbullying. Parenting styles, particularly the degree of psychological control exerted by parents, are considered as significant variables that impact adolescents’ mental health and development. Previous studies suggested that PPC can influence behavioral patterns and psychological states in adolescents, thereby indirectly affecting likelihood of cyberbullying engagements (Barber, 1996). Hence, our study aimed to explore this intricate relationship, focusing on how PPC indirectly influenced adolescents’ involvement in cyberbullying through specific mechanisms.

PPC involves using covert strategies, such as inducing guilt, withdrawing affection and asserting authority, to influence their children’s thoughts and emotions, compelling them to obey parental expectations (Barber, 1996). Numerous studies have found a strong link between high levels of PPC and adolescents’ negative emotions. Adolescents who experience high levels of PPC often exhibit increased symptoms of depression and anxiety (Rogers et al., 2020). Additionally, PPC is also considered a significant risk factor for both internalizing and externalizing problems among adolescents (Davidson et al., 2017; Wouters et al., 2018). Furthermore, PPC is closely related to adolescents’ aggressive behaviors, and it could predict relational and physical aggression in adolescents (Nelson et al., 2013). This may be because, when adolescents feel that their parents have withdrawn their affection, they may adopt maladaptive emotion regulation strategies such as self-blame and catastrophe.(Cui et al., 2014). Therefore, PPC may cause emotional distress and lead to aggressive behaviors in adolescents.

Previous research has found that cyberbullying is significantly associated with depression, anxiety symptoms, and suicidal ideation among adolescents, and may lead to long-term physical and mental health problems (Kowalski et al., 2014; Van Geel et al., 2014; Wang and Ngai, 2020). PPC profoundly influences adolescents’ emotional well-being and behavioral development. Existing studies have indicated that high levels of PPC are closely linked to depression, anxiety, and aggressive behaviors in adolescents (Nelson et al., 2013; Rogers et al., 2020), and such aggressive tendencies may easily extend into the digital realm. Therefore, we hypothesize that PPC may indirectly influence adolescents’ engagement in cyberbullying through certain mediating mechanisms.

Additionally, previous studies have demonstrated that high levels of PPC are significantly correlated with depression and anxiety in adolescents (Keijsers and Poulin, 2013; Rogers et al., 2020; Soenens and Vansteenkiste, 2010), which are often associated with poor sleep quality. When adolescents undergo PPC, they may face heightened emotional stress and negative emotions, which can subsequently impair their sleep quality (Cui et al., 2014; Morris et al., 2002). Furthermore, PPC can hinder the development of healthy sleep habits in adolescents, such as maintaining consistent bedtime routines or engaging in appropriate pre-sleep activities (Blair et al., 2012). Prolonged emotional pressure and irregular sleep patterns may lead to sleep disorders, ultimately resulting in reduced sleep time and sleep quality (Wang et al., 2015). Poor sleep quality affects not only adolescents’ emotional health, but may also contribute to problematic behaviors. Sleep deprivation or poor-quality sleep impairs neurobehavioral functioning and hinders the key behavioral and psychological functions necessary for social interactions (Gregory and Sadeh, 2012; Krizan and Herlache, 2016). Specifically, sleep disturbances can impair adolescents’ cognitive functioning and emotion regulation abilities, making them more likely to resort to aggressive behaviors when facing social conflicts (Kamphuis et al., 2012). Therefore, sleep quality may serve as a mediator in the relationship between PPC and cyberbullying.

Moral disengagement refers to the process by which individuals justify or rationalize their unethical behaviors through cognitive mechanisms (Bandura et al., 1996). PPC can reduce adolescents’ autonomy and self-efficacy, leading to less consideration of actions’ consequences and moral implications, and increasing moral disengagement (Bussey et al., 2015; Bussey et al., 2020). In addition, adolescents in high-pressure environments may become less empathetic and more prone to bullying (Kowalski et al., 2019; Runions and Bak, 2015). One study have also found that PPC has a significant negative effect on the level of moral disengagement among migrant children (Wu et al., 2018). According to the general aggression model, situational factors can influence individuals’ internal cognitive and emotional states, which in turn affect how they interpret events, ultimately leading to aggressive behavior (Anderson and Bushman, 2002). Moral disengagement enables individuals to redefine the nature of their actions, diminish personal responsibility, and reduce empathy toward victims (Bandura et al., 1996). Several studies have shown that moral disengagement is a significant predictor of cyberbullying (Ahmed and Braithwaite, 2005; Kowalski et al., 2014; Olweus, 2013; Smith, 2016). For instance, longitudinal studies highlight the significant predictive role of moral disengagement in both traditional bullying and cyberbullying (Falla et al., 2023; Yang et al., 2022). Furthermore, meta-analytic evidence has also confirmed the critical role of moral disengagement in cyberbullying (Oliveira et al., 2021; Zhao and Yu, 2021). Thus, moral disengagement may mediate the relationship between PPC and cyberbullying.

According to existing theoretical and empirical research, the chained relationship between sleep quality and moral disengagement can be realized through a threefold pathway of cognition-emotion-behavioral control, ultimately contributing to the emergence of cyberbullying behaviors. Sleep deprivation inhibits the functioning of the prefrontal cortex (Davies et al., 2017), weakening an individual’s ability to process complex information, such as interpreting others’ intentions and evaluating behavioral consequences. Adolescents in ambiguous situations are more likely to exhibit hostile attribution bias (Aloia, 2011), misinterpreting others’ actions as aggressive signals. This cognitive bias prompts them to employ moral disengagement mechanisms to rationalize their behavior, thereby reducing feelings of guilt. Poor sleep quality also leads to hyperactivation of the amygdala and emotional dysregulation (Provini and Chokroverty, 2011), resulting in a chronically irritable and anxious state. Negative emotions reduce empathy (Kujawa et al., 2014) and diminish sensitivity to victims’ suffering, enabling individuals to justify aggressive behaviors through strategies such as displacement of responsibility or moral justification (Bandura et al., 1996).

Chronic sleep deprivation depletes physiological resources such as glucose, which directly impairs self-control abilities (Gailliot et al., 2007). When adolescents encounter online conflicts, limited self-regulatory resources make it difficult to suppress aggressive impulses. At this point, moral disengagement functions as a “cognitive shortcut,” quickly resolving the conflict between behavior and moral standards, thus lowering the threshold for behavioral inhibition. Therefore, we proposed that sleep quality and moral disengagement might serve as mediating factors in the relationship between PPC and cyberbullying.

For adolescents, grade level serves as a key moderating variable that significantly influences the relationship between moral disengagement and cyberbullying. Research indicates that as adolescents age, their cognitive development and social experiences gradually mature, leading to changes in their behavioral motivations and social cognitive abilities, which may, in turn, affect the extent of moral disengagement and its influence on cyberbullying. On the one hand, younger students (e.g., elementary school pupils) may not be significantly impacted by moral disengagement in terms of their engagement in cyberbullying. This may be due to their underdeveloped social cognition and insufficient situational judgement (Choudhury et al., 2006). In contrast, for older students (e.g., high school students), with enhanced cognitive abilities and increasing familiarity with social norms, they may choose more covert forms of aggression, such as cyberbullying, to avoid punishment (Chan et al., 2021). Furthermore, current studies have shown that compared to younger children, adolescents aged 14–15 years old show the highest rates of both perpetration and victimization in cyberbullying (Pichel et al., 2021). Therefore, this study aims to further explore whether grade level moderates the effect of moral disengagement on cyberbullying.

**Figure 1:**
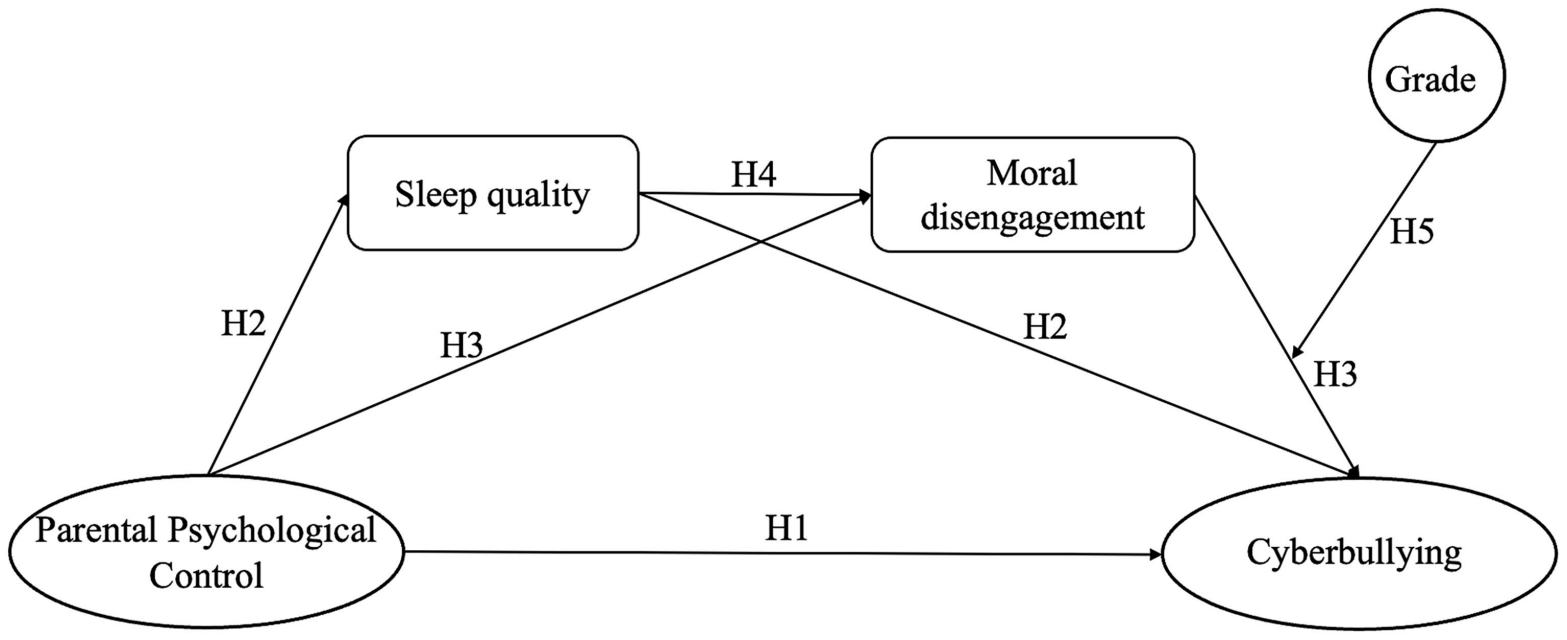
The proposed model about cyberbullying among adolescents in this study. H, hypothesis.

In summary, this study sought to construct a moderated chain mediation model and proposed the following hypotheses (see Figure 1):

*H1*: PPC was positively associated with cyberbullying.

*H2*: Sleep quality mediated the relationship between PPC and cyberbullying.

*H3*: Moral disengagement mediated the relationship between PPC and cyberbullying.

*H4*: Sleep quality and moral disengagement sequentially mediated the relationship between PPC and cyberbullying.

*H5*: Grade level moderated the effect of moral disengagement on cyberbullying.

## 2 Methods

### 2.1 Participants

This study employed convenience sampling to conduct a survey among 688 students from 23 classes in one school. Among them, 357 (51.89%) were boys and 331 (48.11%) were girls. In terms of grade distribution, 313 (45.49%) were in grade 4 (low grade), 293 (42.59%) were in grade 5 (middle grade), and 82 (11.92%) were in grade 6 (high grade). Regarding their origins, 462 (67.15%) were urban students, while 225 (32.70%) were rural students. Concerning only children status, 276 students (40.12%) were only children, whereas 410 (59.59%) had siblings. Regarding parental education levels, 37.57% of fathers and 41.68% of mothers had up to junior high school education, 30.02% of fathers and 29.62% of mothers had high school or vocational degrees, 27.81% of fathers and 26.33% of mothers held bachelor’s or associate degrees, and 4.24% of fathers and 2.38% of mothers had master’s degrees or higher (see Table 1). This study was approved and consented by the Ethics Committee of Wenzhou Medical University in accordance with the Declaration of Helsinki in February 2017, and parents’ informed consent form and students’ assents were both obtained in this study. The survey of this study was conducted between April 2017 and June 2018. During the survey, all students completed the pencil-and-paper questionnaires in quiet classrooms.

**Table 1:** Demographic characteristics of the sample (*N* = 688).

Variable	Category	*n*	%
Gender	Male	357	51.89
	Female	331	48.11
Grade level	Grade 4	313	45.49
	Grade 5	293	42.59
	Grade 6	82	11.92
Residence	Urban	462	67.15
	Rural	225	32.70
Only child	Yes	276	40.12
	No	410	59.59
Father’s education	≤ Junior high school	258	37.57
	High school / vocational	206	30.02
	Bachelor / associate	191	27.81
	Master’s or higher	29	4.24
	Missing	4	0.35
Mother’s education	≤ Junior high school	286	41.68
	High school / vocational	203	29.62
	Bachelor / associate	181	26.33
	Master’s or higher	16	2.38
	Missing	2	0.03

### 2.2 Measures

#### 2.2.1 Parental psychological control

The PPC Scale developed by Barber (1996) and revised by Wang et al. (2007) was used to assess the level of PPC among middle schoolers. The questionnaire consists of 18 items, with ten items related to guilt induction (e.g., “My parents tell me that I should feel guilty when I do not meet their expectations”), five items concerning withdrawal of affection (e.g., “If I do something they do not like, my parents act cold and unfriendly towards me”), and three items addressing authoritative assertion (e.g., “My parents tell me that what they want me to do is best for me, and I should not question it”). The scale uses a 5-point Likert scale ranging from 1 to 5, where students rate their parents based on actual circumstances. The total score, which is the sum of all item scores, indicates the level of PPC, with higher scores reflecting greater control. In this study, the Cronbach’s *α* coefficient for this questionnaire was 0.928.

#### 2.2.2 Sleep quality

Sleep quality was assessed using the Pittsburgh Sleep Quality Index (PSQI) (Buysse et al., 1991). This scale comprises 18 items across seven dimensions: subjective sleep quality, sleep latency, sleep duration, sleep efficiency, sleep disturbances, use of sleeping medication, and daytime dysfunction. Each dimension is rated on a 4-point Likert scale (0–3), with higher scores indicating poorer sleep quality. The total score ranges from 0 to 21, where 0 represents the best sleep quality and 21 represents the worst. In this study, the Cronbach’s *α* coefficient for the PSQI was 0.864.

#### 2.2.3 Moral disengagement

The Moral Disengagement Scale developed by Bandura et al. (1996) and revised by Yang and Wang (2012) was used to measure adolescents’ levels of moral disengagement. This scale includes eight dimensions: moral justification, euphemistic labeling, advantageous comparison, displacement of responsibility, diffusion of responsibility, distortion of consequences, dehumanization, and attribution of blame. It consists of a total of 32 items. A 5-point Likert scale is used, with total scores ranging from 32 to 160; higher total scores indicate higher levels of moral disengagement. In this study, the Cronbach’s α coefficient for this scale was 0.923.

#### 2.2.4 Cyberbullying

The cyberbullying level of participants was assessed using the Cyberbullying Questionnaire (Michelle et al., 2015). This questionnaire consists of nine items, such as “Have you ever insulted peers online or via text messages?” All items are rated on a 5-point scale (1 = never, 5 = always), and the total score is obtained by summing up the scores across all items. Higher scores indicate higher levels of cyberbullying. The questionnaire has demonstrated good reliability and validity in previous studies. In this study, the Cronbach’s α coefficient for this questionnaire was 0.787 (see Table 2).

**Table 2:** Descriptive statistics and reliability of study variables (*N* = 688).

Variable	Subscale/Total	M	SD	Cronbach’s α
Parental psychological control	Total	37.53	13.53	0.928
Sleep quality	Total	4.66	2.59	0.864
Moral disengagement	Total	54.53	16.95	0.923
Cyberbullying	Total	10.47	2.60	0.787

### 2.3 Data analysis

First, SPSS 24.0 was used to perform descriptive and correlation analyses of the study variables and demographic variables. Since the research variables in this study are continuous variables, Pearson correlation was used to explore the correlation between the variables. Then, PROCESS in SPSS was adopted to test moderated chain mediation effects model (Dou et al., 2023; Lv et al., 2025). Parameter estimation was performed using the bootstrap method with 5,000 replicate samples and a confidence interval (CI) with a confidence level of 95%, which indicates that the corresponding effect is significant if the confidence interval (CI) does not include zero, and not significant if the confidence interval (CI) includes zero.

## 3 Results

### 3.1 Common method bias test

As data were collected through self-report measures, there may be concerns regarding common method bias. To assess this, Harman’s single-factor test was conducted. An unrotated exploratory factor analysis yielded 16 factors with eigenvalues greater than 1. The first factor accounted for only 16.50% of the total variance, far below the critical threshold of 40%. Therefore, it can be concluded that serious common method bias is not present in this study.

### 3.2 Descriptive statistics and correlation analysis among study variables

Table 3 shows the results of the correlation analysis between the research variables. All variables showed significant positive correlations. Additionally, *t*-tests revealed significant differences in moral disengagement (*t* = 3.67, *p* < 0.001) and PPC (*t* = 2.15, *p* = 0.032) between gender. One-way analysis of variance found significant differences in PPC (*F*_(7, 680)_ = 2.50, *p* = 0.015), sleep quality (F_(7, 680)_ = 10.58, *p* < 0.001), moral disengagement (F_(7, 680)_ = 3.00, *p* = 0.003), and cyberbullying (F_(7, 680)_ = 2.68, *p* = 0.010) among students of different birth years. Additionally, school type significantly impacted PPC (*F*_(21, 666)_ = 2.10, *p* = 0.003), sleep quality (F_(21, 666)_ = 5.19, *p* < 0.001), moral disengagement (F_(21, 666)_ = 2.63, *p* < 0.001), and cyberbullying (F_(21, 666)_ = 1.68, *p* = 0.029). Given the influence of these variables on the study variables, they were controlled for in subsequent analyses.

**Table 3:** Descriptive statistics and correlation analysis of variables (*n* = 688).

Variable	M	SD	1	2	3	4
1 Parental psychological control	37.53	13.53	—			
2 Sleep quality	4.66	2.59	0.20^**^	—		
3 Moral disengagement	54.53	16.95	0.16^**^	0.25^**^	—	
4 Cyberbullying	10.47	2.60	0.15^**^	0.17^**^	0.20^**^	—

^**^Indicates *p* < 0.01.

### 3.3 Testing the mediation model

The mediation model was tested using Model 6 from Hayes’ Process Macro to examine the impact of PPC on cyberbullying, as well as the mediating roles of sleep quality and moral disengagement. Considering the effects of gender, birth year, and school type on the study variables, these factors were included as control variables in the model.

The results (see Table 4) showed that PPC was significantly correlated with cyberbullying. After incorporating sleep quality and moral disengagement into the model, sleep quality and moral disengagement both mediate the relationship between PPC and cyberbullying.

**Table 4:** Test of the chain mediation effects.

Regression equation		Overall fit coefficients			Significance of coefficients		
Outcome variable	Predictor variable	R	R2	F	*β*	95%CI	*t*
Cyberbullying	Parental psychological control	0.20	0.04	6.83***	0.13	[0.06, 0.20]	3.55^***^
Sleep quality	Parental psychological control	0.34	0.12	22.76^***^	0.18	[0.11, 0.26]	4.79^***^
Moral disengagement	Parental psychological control	0.32	0.10	15.17^***^	0.10	[0.03, 0.18]	2.64^**^
	Sleep quality				0.23	[0.16, 0.31]	6.01^***^
Cyberbullying	Parental psychological control	0.27	0.08	9.21^***^	0.09	[0.02, 0.16]	2.55^*^
	Sleep quality				0.08	[0.01, 0.15]	2.24^*^
	Moral disengagement				0.14	[0.07, 0.21]	4.07^***^

* Indicates *p* < 0.05, ** indicates *p* < 0.01, *** indicates *p* < 0.001; gender, birth year, and school were included as control variables. All variables were standardized.

The results of the mediation analysis (see Table 5) indicated that the total effect was 0.129. The mediating effect of sleep quality was 0.015, the mediating effect of moral disengagement was also 0.015, and the chain-mediated effect (sleep quality → moral disengagement) was 0.006. The Bootstrap 95% confidence intervals for all three indirect pathways did not include zero, indicating that all three mediation effects were statistically significant. These indirect effects accounted for 11.63, 11.63, and 4.7% of the total effect, respectively.

**Table 5:** Mediation effects.

	Effect	SE	95%CI
Total effect	0.129	0.036	[0.057, 0.200]
Direct effect	0.093	0.036	[0.021, 0.164]
Mediating effect of sleep quality	0.015	0.008	[0.001, 0.034]
Mediating effect of moral disengagement	0.015	0.009	[0.002, 0.035]
Chain mediation effect	0.006	0.003	[0.002, 0.012]

The 95% CI refers to the standard error and the lower and upper bounds of the 95% confidence interval estimated using the bias-corrected percentile Bootstrap method for the indirect effects.

### 3.4 Testing the moderation effect

To investigate the moderating role of grade level, Model 87 from Hayes’ Process Macro was utilized. The results (see Table 6) indicated that, after controlling for gender, birth year, and school type, moral disengagement was significantly correlated with cyberbullying, and the interaction term also significantly (*β* = −0.07, *p* < 0.01).

**Table 6:** Test of moderated multiple mediation effects.

Regression equation		Overall fit coefficients			Significance of coefficients		
Outcome variable	Predictor variable	R	R2	*F*	*β*	95%CI	*t*
Sleep quality	Parental psychological control	0.34	0.12	22.76***	0.18	[0.11, 0.26]	4.79***
Moral disengagement	Parental psychological control	0.32	0.10	15.17***	0.10	[0.03, 0.18]	2.64**
	Sleep quality				0.23	[0.16, 0.31]	6.01***
Cyberbullying	Parental psychological control	0.29	0.08	7.82***	0.10	[0.03, 0.17]	2.67**
	Sleep quality				0.08	[0.01, 0.15]	2.20*
	Moral disengagement				0.14	[0.08, 0.21]	4.12^***^
	Grade				−0.25	[−0.53, 0.04]	−1.72
	Moral disengagement *grade				0.07	[0.003, 0.14]	2.04^*^

*Indicates *p* < 0.05, ** indicates *p* < 0.01, *** indicates *p* < 0.001; gender, birth year, and school were included as control variables. All variables were standardized.

**Figure 2:**
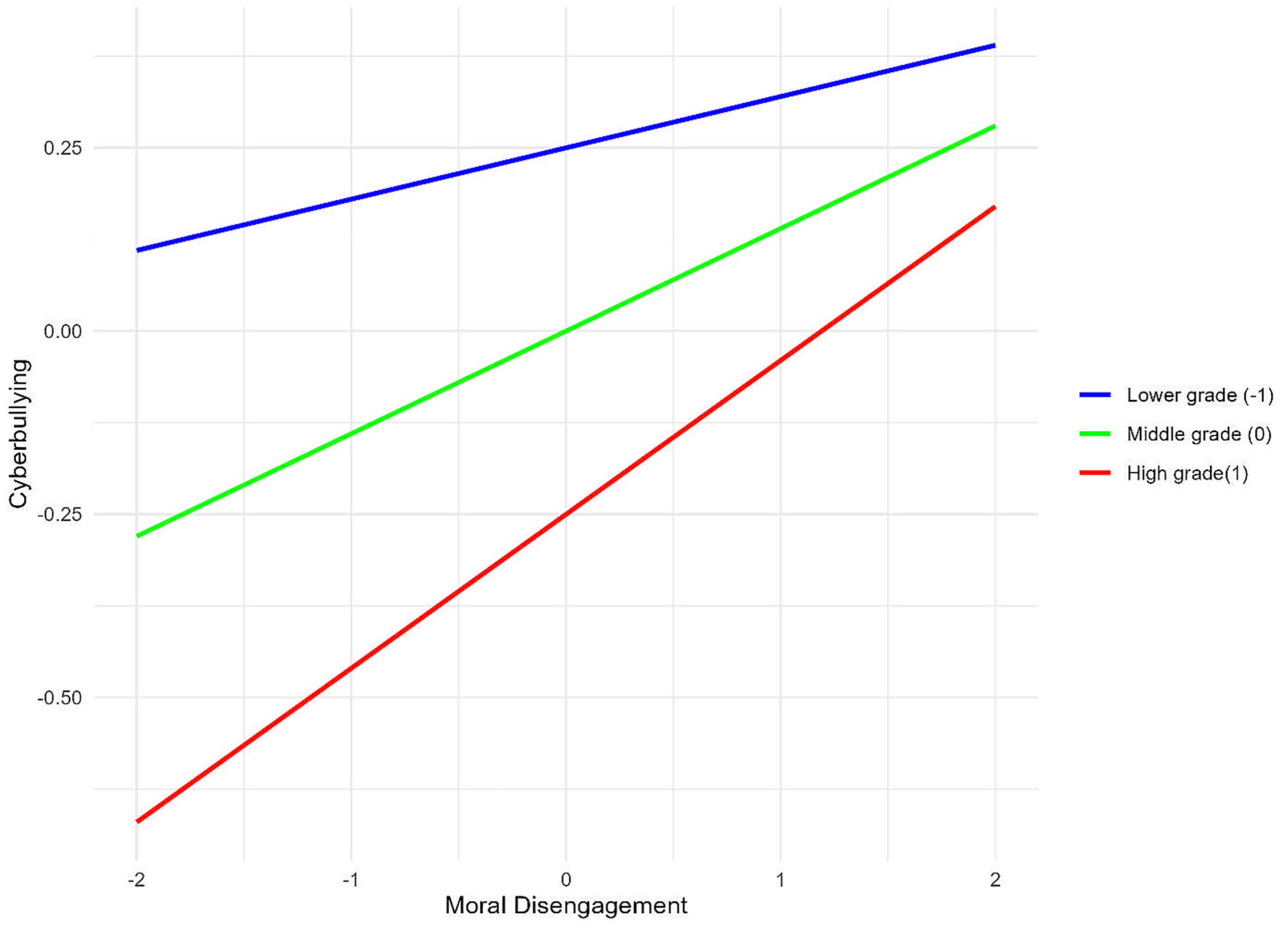
The moderating role of grade between to nature between moral disengagement and cyberbullying.

To better illustrate the moderating effect of grade level, simple slope analysis was conducted. Figure 2 shows that for lower-grade participants (elementary school students), the effect of moral disengagement on cyberbullying is not significant (simple slope = 0.07, *p* = 0.154). In contrast, for higher-grade participants (high school students), the effect of moral disengagement on cyberbullying is significant (simple slope = 0.21, *p* < 0.001).

## 4 Discussion

This study explored the impact of PPC on cyberbullying from a family environment perspective, revealing the mediating roles of sleep quality and moral disengagement in this process. Additionally, it found that moral disengagement affected cyberbullying differently by grade level, with high school students showing a stronger effect than elementary students These findings indicated that parenting styles might play a crucial role in cyberbullying and highlight the critical influence of adolescent development, social cognitive maturity and emotional regulation ability in the modern online environment. Our study provided new insights into the complexity of cyberbullying and offers theoretical support for the design of personalized preventive measures in education and mental health interventions.

The results showed that PPC significantly was correlated with adolescents’ cyberbullying behavior, consistent with our hypothesis. This finding suggested that parental emotional manipulation undermines children’s autonomy which led to online aggression. Specifically, PPC may exhibit negative cognitive patterns in adolescents, making them more likely to express emotions through violence or bullying when facing social conflicts or emotional stress. This pattern affects adolescents’ responses to bullying, including covert cyberbullying. Research also indicates that PPC not only influences adolescents’ involvement in traditional bullying but also significantly increases their tendency toward cyberbullying (Liang et al., 2024). This indicates that PPC influences adolescents’ involvement in both traditional and cyberbullying. These results suggested that parental emotional manipulation has profound effects on adolescents in both real-world and virtual environments.

The results of this study indicated that both sleep quality and moral disengagement partially mediate the relationship between PPC and cyberbullying. This finding revealed the complex mechanisms through which PPC influences adolescents’ engagement in cyberbullying behavior. Specifically, PPC may affect adolescents’ sleep quality through several possible pathways. The emotional pressure caused by PPC may lead to increased anxiety and worry among adolescents at night, thereby disrupting the process of falling asleep (Johnson et al., 2000; Sarchiapone et al., 2014; Schredl et al., 2009).

Moreover, the mediating role of sleep quality aligns with the cognitive-emotional depletion model (Gregory and Sadeh, 2012). According to this model, sleep deprivation significantly impairs adolescents’ cognitive and emotional regulation abilities. Specifically, insufficient sleep causes difficulties with concentration and memory, and negatively affects emotional regulation and decision-making (Lo et al., 2016). These cognitive and emotional deficits may weaken adolescents’ ability to process complex social information, making them more prone to misinterpret or misjudge others’ intentions during online interactions. For example, lack of sleep may cause adolescents to overreact to aggressive online messages or misinterpret social behaviors, increasing cyberbullying risk (Lund et al., 2010; Shin et al., 2005). Poor sleep quality may also lead to emotional instability, leading to hostile online behaviors and more cyberbullying.

The mediating effect of moral disengagement supports Bandura’s et al. (1996) theory of moral disengagement (Bandura et al., 1996), which posits that individuals justify or rationalize their unethical behaviors through various cognitive mechanisms. PPC, especially conditional expressions of love and care, can increase adolescents’ likelihood of using moral disengagement by affecting their self-concept and emotional stability (Qi, 2019; Wang et al., 2019). When parental love and care are conditional, adolescents often feel that they must meet parental expectations to receive love and approval. This emotional insecurity may lead to immoral behaviors and make them use moral disengagement to reduce guilt (Gao et al., 2023). For instance, adolescents might rationalize the victim’s behavior as “deserving punishment” or believe that “they are just teaching someone a lesson” to justify their bullying actions (Dishion and Bullock, 2001; Gentile et al., 2014). These findings suggest that this pattern of interaction between parents and children provides a psychological basis for adolescents’ engagement in cyberbullying. PPC increases adolescents’ likelihood of cyberbullying through moral disengagement (Ching and Wu, 2018; Laird and Frazer, 2020).

The research findings indicate that the chain pathway from sleep quality to moral disengagement is statistically significant. Specifically, sleep problems indirectly exacerbate adolescents’ aggressive behaviors by impairing moral cognition. This finding aligns with the previous work (Krizan and Herlache, 2016), which suggests that declining sleep quality affects individuals’ emotional regulation and social cognitive abilities, thereby intensifying aggressive tendencies. Sleep deprivation affects the prefrontal cortex, impairing adolescents’ decision-making and increasing susceptibility to hostile attribution bias and misinterpretation of intentions (Crone and Dahl, 2012). Sleep-deprived adolescents often misread social cues as hostile, leading to actions like cyberbullying (Nagata et al., 2023). Moreover, chronic sleep problems may reduce adolescents’ empathy (Guadagni et al., 2014), further reinforcing their tendency toward moral disengagement (Wang et al., 2024). In high-pressure environments, particularly when experiencing prolonged emotional stress and insufficient sleep, young people may become less sensitive to others’ emotions (Minkel et al., 2011). Research by Cui et al. (2014) indicates that both PPC and adolescents’ emotional regulation abilities are influenced by sleep quality. Sleep deprivation impairs adolescents’ empathetic perception and social cognition, increasing bullying behavior. So, adolescents may use moral disengagement to rationalize aggression, shifting blame to victims, causing cyberbullying (Cui et al., 2014).

The results also reveal a significant moderating role of grade level in the relationship between moral disengagement and cyberbullying. Specifically, the effect of moral disengagement on cyberbullying is stronger among high schoolers than pupils, while no significant effect is observed. This phenomenon may be closely related to the more complex social pressures faced by older students, such as academic competition, peer pressure, and intensified social comparisons. As adolescents age, their social and emotional experiences become complex, demanding more from their emotional and cognitive regulation (Yang et al., 2022). Studies suggest that, as they progress through high school, students gradually demonstrate reduced flexibility in moral reasoning. This makes them more likely to use moral disengagement to justify unethical behaviors in cyberbullying-related situations (Scharkow et al., 2014). Additionally, older students often understand social norms and legal consequences better, leading them to prefer covert aggression like cyberbullying to avoid direct conflict (Zhao and Yu, 2021). Furthermore, as they grow older, adolescents increase both the frequency and proficiency with which they use online platforms (Álvarez García et al., 2018). High school students usually begin using social media and other digital platforms at an earlier stage than younger students, resulting in higher exposure to cyberbullying opportunities (Calmaestra et al., 2020). Given their greater presence and influence on these platforms, older students may be more inclined to engage in cyberbullying as a way of social interaction. This pattern indicates that older adolescents use moral disengagement to cope with social stress and often bully on familiar platforms (Pichel et al., 2021).

This study innovatively explores the chained pathway involving PPC, sleep quality, and moral disengagement, thereby expanding existing theoretical models on how parenting styles influence cyberbullying. It offers a novel perspective for understanding adolescent cyberbullying behaviors and highlights the intrinsic connections among PPC, sleep quality, and moral disengagement. The findings suggested that PPC indirectly promotes online aggressive behaviors through the sequential pathway of poor sleep quality and heightened moral disengagement, as supported by our measured constructs. Specifically, parents’ emotionally manipulative behaviors may lead to maladaptive emotion regulation strategies (Kunz and Grych, 2013), which intensify negative emotions and contribute to behavioral problems (Bean et al., 2003). This manipulation and reduced responsibility increase adolescents’ moral disengagement, leading to more aggressive online behaviors (Kim and Kim, 2013).

The significance of this study lies in its contribution to extending existing theoretical models for understanding the mechanisms underlying cyberbullying, particularly within the context of family environment and parenting styles. By revealing the indirect effects of PPC on adolescents’ engagement in cyberbullying, this research enriches our understanding of how such behaviors develop. It highlights the potential role of sleep quality in moral cognitive development and introduces a psychophysiological perspective into the study of moral disengagement. PPC may lead adolescents to adopt maladaptive emotion regulation strategies, thereby intensifying negative emotions and contributing to behavioral problems (Morris et al., 2007; Steinberg and Morris, 2001). Therefore, parents should avoid using PPC and instead adopt supportive parenting approaches. For instance, emotional validation can help children better understand and manage their emotions, thus reducing emotional distress and behavioral issues (Scharf and Goldner, 2018). So, interventions for adolescent cyberbullying should focus on family environment, particularly PPC improvements. First, parents should learn healthier parenting to reduce emotional manipulation and psychological pressure, avoiding tactics like inducing guilt or withdrawing affection to improve adolescents’ emotional regulation and self-efficacy. Second, improving adolescents’ sleep quality should be a key objective in intervention programs. Research shows that sleep quality is closely linked to emotional well-being; under high-pressure conditions, sleep disturbances may exacerbate emotional distress and cognitive distortions. Therefore, both families and schools support to help adolescents establish healthy sleep routines. Regular assessments of sleep quality can aid in early detection of potential problems and allow timely interventions (Dahl and Lewin, 2002; O'Brien, 2009). Moreover, interventions targeting moral disengagement are important especially in online environments. Educators and parents can improve adolescents’ moral judgment by promoting responsibility and curbing moral disengagement. In addition to focusing on individual-level interventions, schools and policymakers should also work toward strengthening campus-based cyberbullying prevention systems. This includes enhancing cybersecurity education, establishing clear codes of conduct, and implementing effective reporting and response mechanisms (Hinduja and Patchin, 2014).

However, this study has several limitations. First, the data used in this study were cross-sectional in nature. While they allow for the examination of associations among variables, they do not permit causal inferences. Future studies are needed to clarify the causal relationships between PPC, sleep quality, moral disengagement, and cyberbullying. Second, the data were primarily collected through self-report questionnaires, which may be subject to social desirability bias. Adolescents might underreport their involvement in cyberbullying or overattribute their behavior to external factors. Future research could incorporate multiple data collection methods—such as observational studies or interviews—to improve the reliability and validity of the findings. Thirdly, this study was conducted in one school which might compromise the generalizability of the findings. Fourth, the distribution of participants across grade levels was uneven, with only 82 students (11.92%) representing the higher grade group. This limited subsample may introduce potential bias in the moderation analysis, and future studies should recruit larger and more balanced samples across grade levels to further validate this effect. Future research should consider selecting sample from different schools in different provinces. Finally, in this study, the PSQI has not yet been formally validated in the general Chinese paediatric population and that our findings should be interpreted with this caveat.

## 5 Conclusion

This study integrated the chained pathway of PPC, sleep quality, and moral disengagement to reveal the significant roles of parenting style, adolescent sleep health, and moral cognition in cyberbullying. The findings emphasized the importance of family parenting practices, adolescents’ moral reasoning and sleep health in preventing and addressing cyberbullying. Interventions to improve PPC, adolescent sleep quality, and moral judgement can reduce cyberbullying and promote healthy development.

## Data Availability

The original contributions presented in the study are included in the article/supplementary material, further inquiries can be directed to the corresponding authors.
